# Determinants of Acceptance of Weight Management Applications in Overweight and Obese Individuals: Using an Extended Unified Theory of Acceptance and Use of Technology Model

**DOI:** 10.3390/nu14091968

**Published:** 2022-05-08

**Authors:** Alexander Bäuerle, Anna-Lena Frewer, Vanessa Rentrop, Lynik Chantal Schüren, Marco Niedergethmann, Julia Lortz, Eva-Maria Skoda, Martin Teufel

**Affiliations:** 1 Clinic for Psychosomatic Medicine and Psychotherapy, University of Duisburg-Essen, LVR-University Hospital Essen, 45147 Essen, Germany; anna-lena.frewer@uni-due.de (A.-L.F.); vanessa.rentrop@uni-due.de (V.R.); eva-maria.skoda@uni-due.de (E.-M.S.); martin.teufel@uni-due.de (M.T.); 2 Center for Translational Neuro- and Behavioral Sciences (C-TNBS), University of Duisburg-Essen, 45147 Essen, Germany; 3 Department of Surgery, Alfried-Krupp Hospital Essen, 45131 Essen, Germany; lynik.schueren@krupp-krankenhaus.de (L.C.S.); marco.niedergethmann@krupp-krankenhaus.de (M.N.); 4 Department of Cardiology and Vascular Medicine, West-German Heart and Vascular Center Essen, University of Duisburg-Essen, 45147 Essen, Germany; julia.lortz@uk-essen.de

**Keywords:** eHealth, UTAUT, BMI, performance expectancy, effort expectancy, social influence

## Abstract

Overweight and obesity carry a tremendous burden in terms of physiological and psychological comorbidities. There is a great variety of weight management applications to support weight reduction, but a systematical analysis of individuals’ needs and requirements to adopt sustaining lifestyle changes is missing so far. This study aimed to assess the acceptance of such applications and its underlying predictors in individuals with overweight/obesity. A cross-sectional study was conducted, including 439 overweight/obese individuals. Health-related internet use and acceptance of weight management applications were examined using a modified questionnaire based on the Unified Theory of Acceptance and Use of Technology (UTAUT). The general acceptance of weight management applications was high, with significant age differences. Compared to older individuals, younger ones showed a higher acceptance. BMI was not significantly associated with acceptance. Besides psychometric data and eHealth-related data, performance expectancy, effort expectancy, and social influence proved to be significant predictors for acceptance. The total variance explanation provided by the extended UTAUT model was 61.2%. The knowledge of the influencing factors on acceptance might be useful in developing, optimizing, and establishing weight management applications. For determining acceptance and its predictors of weight management applications, the UTAUT model is a valid approach.

## 1. Introduction

The treatment of overweight and obesity is becoming increasingly important, as the global incidence shows an impressive incline over the last years [1]. In 2016, 1.9 billion people of the adult population were overweight, including 13% (650 million) being obese [1]. The average proportion of overweight people in 2019 showed a gender difference in the EU [2]. In detail, men (60%) were significantly more often overweight than women (46%); in Germany, these proportions were slightly higher (men: 61%, women: 47%) [2]. Given the currently rising trend, the World Health Organization (WHO) “estimates that by 2025, approximately 167 million people—adults and children—will become less healthy because they are overweight or obese” [3]. Globally, obesity and overweight were estimated to cause 3.4 million deaths, 3.9% of years of life lost, and 3.8% of disability-adjusted life years [4].

Overweight and obesity have been associated with many other mental and physical comorbidities, putting affected individuals at special risk [5]. The most common comorbid physical diseases are cardiovascular disease, type 2 diabetes, fatty liver disease, various types of cancer, obstructive sleep apnea, and musculoskeletal disorders [6]. All these physical diseases lead to a reduced quality of life and are often associated with many disadvantages for these individuals [7]. In addition to physical comorbidities, obese people also frequently suffer from mental disorders. Previous research has illuminated that obesity is very commonly associated with a significant increase in lifetime diagnoses of major depression, bipolar disorder, and panic disorder or agoraphobia [8]. The comorbidities evidenced to date in obese individuals highlight both the complexity and the immediate need for effective and sustained weight management.

### 1.1. Weight Management Applications

Paradoxically, the rates of obese individuals continue to rise even though there is now an ever-increasing wealth of weight management programs and products that attempt to reduce overweight in society [9]. Especially since the rate of obese individuals continues to increase despite more and more disease-associated offers, the addition of novel treatment regimes, including eHealth technologies, takes on a central role. eHealth technologies positively influence health awareness and the adoption and maintenance of healthy lifestyle habits. The continuous adoption into a regular care setting would majorly impact individuals and healthcare practitioners, too [10]. Conventional weight loss and health improvement programs for obese individuals, which focus primarily on healthy food and physical activity, have been widely researched to be effective [11,12]. However, it is important to note that such face-to-face interventions are often very time-consuming, costly, and resource-intensive, making them, in many ways, very impractical for individuals [13]. Moreover, an intensive weight management program in face-to-face contact usually requires a support period of more than one year [13].

The development and establishment of digital evidence-based weight management applications could be a cost-effective, novel, and widely accessible way for people with overweight and obesity to manage their weight successfully [14]. They may be particularly convenient for obese individuals, who often face barriers (e.g., due to limited mobility) to attending face-to-face therapy for extended periods of time. Mobile applications have the huge advantage of being available to individuals everywhere and offer a very promising alternative to face-to-face contact through self-monitoring and tailored feedback [15]. To establish such applications, clinical intervention strategies can be translated into digital approaches (e.g., accessible via smartphones or tablets) [12].

There is already existing research that supports the success of using weight management applications in obese individuals [16]. A review illuminated that all technological interventions included demonstrated a beneficial impact of text messaging or smartphone applications for reducing physical inactivity and/or weight [16]. Another meta-analysis by Liu et al. in 2015 examined the association between the use of mobile phone interventions and patient weight change. The results indicate that the mobile phone intervention was associated with significant changes in body weight and body mass index compared with the control group [17]. Overall, the use of weight management applications focused on successful weight loss, improved physical activity, and improved eating behaviors demonstrated superior efficacy compared with no-intervention control groups [18]. In a meta-analysis, 39 studies about mobile health applications were analyzed in terms of participant characteristics, effective technology components, additional treatments, impact on health-related behaviors, and treatment efficacy. Ultimately, it highlighted the high level of patient satisfaction, usability, and impact on weight reduction [19]. The results also suggest that satisfactory treatment adherence, associated weight loss, and maintenance are achieved via a high level of engagement with mobile health applications [19]. These findings provide evidence that digital intervention may be a useful tool for promoting weight loss among overweight and obese adults.

In order to create and implement innovative eHealth interventions for overweight and obesity, solutions tailored to the needs and requirements of the target group are needed more than ever. Above all, there is the actual question of what motivates individuals to use such technologies. The first and most prominent factor is the acceptance of such technology [20]. Acceptance can be operationalized as the Behavioral Intention (BI) to use such technology. To assess the acceptance and usage behavior of different smartphone applications among individuals, the Unified Theory of Acceptance and Use of Technology (UTAUT) has proven to be a useful approach in several studies [21,22,23,24].

### 1.2. The Unified Theory of Acceptance and Use of Technology Mode

According to the UTAUT model, direct determinants that can capture the acceptance (intention to use) of any kind of technology (e.g., weight management applications) are the following three predictors: performance expectancy (PE), effort expectancy (EE), and social influence (SI). PE describes to which degree an individual believes that it will benefit from using the offered technology. EE is defined as the degree of ease associated with the use of the technology and SI specifies the degree to which an individual perceives that important others, e.g., family or friends, believe he or she should use the technology [25]. To date, research has already addressed the acceptance of eHealth interventions in different patient groups several times and used the UTAUT model to capture the various possible predictors [21]. The studies concluded that the acceptance of eHealth interventions in different patient groups is mostly moderate [22,24,26]. In addition, the three UTAUT variables (PE, EE, SI), which are also assessed in this study, were found to be significant predictors of the acceptance of eHealth interventions in previous research independent of the patient group and various sociodemographic variables [22,24,26]. Thereby, PE was identified as the key predictor of acceptance in the recent studies, with negative outcome expectations predicting lower acceptance of the eHealth intervention [23,24]. Thus far, there is no concrete research on weight management applications using the UTAUT model and only limited research related to the acceptance of other smartphone applications in the health sector in general.

A previous study used the UTAUT model to assess the acceptance of applications designed to increase physical activity behavior among college students in China [27]. The analysis of data from 1704 students showed that all three UTAUT core predictors (PE, EE, SI) were positively associated with the acceptance of physical activity applications after adjusting for background variables. Furthermore, PE had stronger associations with the acceptance of those applications among those whose BMI was beyond normal compared with those whose BMI was within the normal range [27]. Another study in 2019 focused on the predictors that influence the acceptance of diabetes management applications. To assess the possible predictors of acceptance, the UTAUT model was used. The results proved that PE and SI had the strongest effects on the acceptance, wherefore they are the most critical determinants of the acceptance of diabetes management applications [28]. In a study aiming to assess the acceptance of mobile health applications for disease management in patients with multiple sclerosis, generally, moderate acceptance was found, with lower acceptance among patients with no eHealth experiences and higher acceptance among patients with regular usage of diabetes management applications. PE and SI were found to be significant predictors of acceptance of mobile health applications for disease management [28].

### 1.3. Objectives

In general, it is observed that most patient groups have moderate to high attitudes towards mobile health applications and perceive them to be helpful in many cases. However, in order to increase the acceptance and use of such applications, it is essential to determine the predictors of acceptance. These predictors can be useful in health app development and implementation. To date, there have been no studies addressing the predictors of acceptance of weight management applications among overweight and obese individuals by using validated and established research models. Therefore, this study firstly aims to assess acceptance, differences in acceptance concerning sociodemographic and medical data, and ultimately the determination predictors of acceptance. It also examines whether an extended UTAUT model explains a higher variance in acceptance than the original UTAUT model. Previous research examined acceptance and various predictors in other patient groups, leading to propose the following assumptions for this study:

1. The general acceptance of weight management applications among overweight and obese individuals is moderate.
2. It is expected that there will be differences found in the acceptance of weight management applications depending on gender, age, degree of obesity, and previous experience with eHealth interventions.
3. There is a positive relation between PE, EE, and SI (UTAUT factors) and the acceptance of weight management applications.
4. In addition to the UTAUT factors, it is hypothesized that sociodemographic, medical, and eHealth-related data may be determinants for acceptance of weight management applications.
5. A significant difference in variance explanation of acceptance is expected when comparing the original UTAUT model and an extended UTAUT model, including additional predictors.

The results of this study could be crucial for the development and implementation of beneficial weight management applications for overweight and obese individuals, as by highlighting the predictors of acceptance, it is more feasible to develop tailor-made applications. The gained knowledge will significantly impact the implementation and therefore the clinical routine.

## 2. Materials and Methods

### 2.1. Participants and Procedure

Participants in this study were recruited at the Obesity Center of Alfried Krupp Hospital and via official topic-related social media platform groups, such as Facebook, from 8 July 2020 to 1 February 2021. The selection criteria were an age of 18 years or above, a good command of the German language, Internet access, and a BMI > 25 kg/m^2^. Electronic informed consent was obtained before the survey began, and participation was completely anonymous and voluntary. The average processing time to take the online questionnaire was around 18 min. No financial compensation was offered. Of 996 participants starting the survey, 643 (64.6%) completed it. A total of 95 participants were excluded from the 643 participants due to being normal or underweight. In order to improve data quality, the slowest and fastest 10% (completion time of the assessment) of all participants were excluded. Such data cleaning aims to remove outliers (related to processing time) that indicate careless and unreliable study participation [29]. Furthermore, such outliers harbor the risk of severe bias in statistical analyses, e.g., some descriptive measures are not robust to outliers [29,30]. This resulted in a complete data set of 439 participants who fulfilled the criteria. The survey was conducted in accordance with the Declaration of Helsinki, and the Ethics Committee of the Essen Medical Faculty (19-89-47-BO) agreed to the study protocol.

### 2.2. Assessment Instruments

The online survey contained items on sociodemographic and medical information, validated assessment instruments, a modified version of the UTAUT questionnaire, and items on eHealth-related data.

Sociodemographic data were assessed, including age, sex, marital status, educational level, and occupational status. Moreover, individuals were asked about their weight, height, and mental and physical illnesses.

Eating Disorder Examination—Questionnaire 8 (EDE-Q8): The EDE-Q8 is a short version of the EDE-Q and comprises four subscales (restraint, eating concern, shape concern, weight concern). It consists of five items assessing eating disorder psychopathology in the past 28 days on a 7-point Likert scale ranging (from 0 = not one day to 6 = every day) and three items assessing the occurrence and frequency of core eating disorder behavior on a scale (from 0 = never to 6 = every time) [31]. Cronbach’s α in this study was 0.84, which indicates a good internal consistency.

Eating Disorder Inventory-2—Bulimia (EDI-2-B): The EDI-2-B consists of seven items assessing symptoms of bulimia (especially binge eating) on a 6-point Likert scale (1 = never to 6 = always). The sum score has a minimum of 7 points and a maximum of 42 points [32]. Cronbach’s α in this study was 0.80, indicating good internal consistency.

Patient Health Questionnaire-8 (PHQ-8): The PHQ-8 consists of eight items assessing depressive symptoms over the past two weeks. The answers are given on a 4-point Likert scale (ranging from 0 = not at all to 3 = nearly every day). The cut-off for major depression symptoms is a sum score > 10 [33]; the internal consistency was high with a Cronbach’s α of 0.86.

To assess the eHealth-related data, several items measuring Internet use, Internet anxiety, attitudes towards and experiences with online interventions, and confidence with social media were used. These items were measured on a 5-point Likert scale (1 = totally disagree to 5 = totally agree).

The UTAUT questionnaire was used to assess the acceptance of weight management applications. It consists of 12 items, and answers are given on a 5-point Likert scale (ranging from 1 = totally disagree to 5 = totally agree). Respectively, three items measure acceptance, which is operationalized as the intention to use (BI) and its underlying predictors, namely SI, PE, and EE. In this study, Cronbach’s α values were 0.89 for acceptance (BI), 0.83 for SI, 0.86 for PE, and 0.83 for EE, proving high internal consistency of all scales. See Supplemental Materials I for a translated version of the UTAUT items used in this study.

### 2.3. Data Analysis

Before performing any statistical test, the relevant prerequisites have always been tested. The statistical analyses were performed using SPSS Statistics version 26 (IBM, New York, NY, USA) [34]. In the first step, internal consistencies for the different psychometric questionnaires, and descriptive statistics were calculated. Moreover, in accordance with previous research, the acceptance (BI) was categorized as low (1–2.34), moderate (2.35–3.67), and high (3.68–5) [35]. Sum scores, as well as mean scores for the scales EDI-2-B, EDE-Q8, and PHQ-8, were computed. Four age categories were formed prior to analysis: (1) 18–34, (2) 35–44, (3) 45–54, and (4) > 55. The BMI of the participants was calculated by dividing the body weight by the height in meters squared. In addition, BMI categories were formed to determine the degree of obesity. In detail, this study included the following BMI categories: 25.0–29.9 pre-obesity (overweight), 30.0–34.9 obesity grade I, 35.0–39.9 obesity grade II, and ultimately, above 40 obesity grade III [36]. The means of acceptance (BI) were compared between groups regarding sociodemographic and medical data with *t*-tests and ANOVAs to include variables with multiple categories. The level of significance was set at α = 0.05 (two-sided test). The mean comparisons were followed by post hoc tests, which included a α correction using the Bonferroni method. Considering the current sample size (*N* = 439), a normal distribution (see central limit theorem) in the variables was assumed [29], so parametric tests were used.

RStudio version 4.0.2 (RStudio PBC, Boston, MA, USA) [37] was used for the data analysis. The model of acceptance was tested by using multiple hierarchical regression. The following variables were included blockwise: (1) sociodemographic and medical data, (2) psychometric data, (3) eHealth-related data, and (4) UTAUT predictors. In the last step, the extended UTAUT model was tested against the original UTAUT model (only including the three core predictors of PE, EE, SI) in a model comparison via ANOVA. No multicollinearity could be detected since variance inflation factor (VIF) values for testing multicollinearity were all VIF < 2 [38]. The qq-plots of the residuals were visually inspected and showed no signs of violations against normality, so normal distribution of the residuals can be assumed. Homoscedasticity was proven based on a scatter plot of the standardized residuals and the adjusted predicted values.

## 3. Results

### 3.1. Sociodemographic and Medical Data

A total of 439 participants was predominantly obese (85.0%) and less overweight (15.0%). Most of the individuals were female (89.3%), aged between 45–54 (31.0%), and married (52.4%). In the present sample, most participants suffered from obesity grade III (53.3%), and 159 of all participants also had a mental disorder in addition to being overweight or obese (36.2%). For all details, Table 1 shows the aggregated characteristics of the present sample.

### 3.2. Differences in Acceptance of Weight Management Applications

The general acceptance of weight management applications in overweight and obese people was high, with a mean of 3.83 (*SD* = 0.93). Considering the acceptance categories from low to high, a total of 129 (29.4%) people showed low acceptance, 195 (44.4%) people moderate acceptance, and a total of 115 (26.2%) people reported high acceptance of weight management applications. Acceptance of weight management applications differed significantly between age categories (*F*_3, 435_= 5.77 *p* = 0.001). Post hoc tests showed that significant difference is between age groups two and three (*p* < 0.001) and between age groups two and four (*p* = 0.043), with the highest acceptance among persons aged 35–44. No differences in acceptance regarding sex, BMI groups, outpatient psychotherapy, educational status, occupational status, and suffering from a mental disorder were detected. A report summarizing the results found with regard to differences in acceptance is presented in Table 2.

### 3.3. Predictors of Acceptance of Weight Management Applications

To evaluate predictors of acceptance of weight management applications, a hierarchical linear regression analysis was conducted. In the first step, sociodemographic (i.e., occupational status, age, gender) and medical (i.e., BMI, mental disorder) data were analyzed. Together, all five variables explain 1.94% of the variance in the acceptance of weight management applications, *F*_5, 433_ = 1.72, *p* = 0.130. No significant predictors could be observed.

In the second step, psychometric data were added to the analysis. Contrary to step one, step two was significant, *R^2^* = 0.05, *F*_8, 430_ = 2.97, *p* = 0.003. The explanation of variance in step two was small [39]. Step two accounted for 5.23% of the acceptance of weight management applications. However, none of the included variables predicted acceptance significantly.

In step three, eHealth-related data were added to the model. As well as step two, step three was significant, *R^2^* = 0.09, *F*_12, 426_ = 3.57, *p* < 0.001. In detail, step three accounted for 9.14% of the variance in the acceptance of weight management applications. The explanation of variance in step three was small [39]. In detail, the following variables showed a significant prediction in step three: EDE-Q8 Total (*B =* 0.11, 95% *CI* (0.03, 0.19)*, p =* 0.010), Internet anxiety (*B =* −0.13, 95% *CI* (−0.25, −0.00)*, p =* 0.047), and confidence with social media (*B =* 0.13, 95% *CI* (0.03, 0.22), *p =* 0.001).

Finally, in step four, the UTAUT predictors (EE, PE, SI) were added to the model (overall model). Step four was significant, *R^2^* = 0.61, *F*_15, 423_ = 44.5, *p* < 0.001. In detail, step four accounts for 61.2% of the explained variance in the acceptance of weight management applications. Consequently, the UTAUT predictors’ effect explains 52.06% of the variance in the acceptance of weight management applications. The explanation of variance in step four is large [39]. In detail, the following variables showed a significant prediction: UTAUT PE (*B =* 0.23, 95% *CI* (0.15, 0.31)*, p <* 0.001), UTAUT EE (*B =* 0.43, 95% *CI* (0.34, 0.51)*, p <* 0.001), and UTAUT SI (*B =* 0.40, 95% *CI* (0.31, 0.49)*, p <* 0.001). Consequently, only the UTAUT predictors (PE, EE, and SI) are positively associated with the acceptance of weight management applications in the overall model.

For a summary of this hierarchical regression analysis for variables predicting acceptance of weight management applications, see Table 3.

### 3.4. Extended UTAUT vs. Original UTAUT

A model comparison between the original UTAUT model and the extended UTAUT model was conducted. The original UTAUT model only includes the three core predictors EE, PE, and SI [20,21]. The explained variance in acceptance of weight management applications of the original UTAUT model was 60% (*R^2^* = 0.60, *F*_3, 435_ = 218, *p* < 0.001). The extended UTAUT model, including several sociodemographic, medical, psychometric, and eHealth-related data [35,40] besides the three core predictors, reached 61.2% in explained variance (*R^2^* = 0.61, *F*_15, 423_ = 44.5, *p* < 0.001). However, since this model offers only 1.2% more explanation of variance in the acceptance of weight management applications than the original model, the model comparison via ANOVA is not significant, *F*_12, 423_ = 1.09, *p* = 0.368. Following this, there is no significant difference in variance explanation in the acceptance of weight management applications when the original UTAUT model is compared to an extended UTAUT model that includes additional variables.

## 4. Discussion

Overweight and obesity have proven to be an increasing phenomenon in society worldwide [1,2,3]. Although online offers of training programs to reach and maintain a healthy weight are booming and quite popular [11], they do not prevent the rise of overweight and obesity. This is a fatal trend since overweight and obesity are apparent risk factors for premature deaths and disability adjustments [4]. Therefore, weight management applications have to be optimized. This is the first study that aims to assess the acceptance of weight management apps among individuals with overweight and obesity and to examine the underlying predictors that influence the intention to use such apps.

Overall, the general acceptance of weight management applications in overweight and obese people was high (44.4% of the sample showed moderate acceptance, while 26.2% showed high acceptance). This fits with patients considering such applications (i.e., mobile health applications) as “satisfactory, easy to use, and helpful in the pursuit of weight loss goals” [18]. PE, EE, and SI were significant predictors of acceptance in the overall model. The overall model provided 61.2% of explained variance in acceptance of weight management applications. Additionally, eating disorder symptoms, confidence with social media, as well as Internet anxiety were critical predictors of acceptance—but only without the UTAUT predictors’ influence.

In detail, the acceptance could be predicted by age. While people aged 35–44 years showed the highest acceptance and those aged 18–34 years showed the second-highest acceptance, people over 45 years were the least accepting. In previous research, this age effect is not unknown since it is assumed, for example, that individuals over the age of 50 prefer conventional media (e.g., home telehealth services) [41]. A possible explanation for the differing level of acceptance regarding the participants’ age could be quite intuitive. Digitalization is a phenomenon that arose in the 1950s [42]. However, for over 40 years, it was still in its infancy and had to be developed further [42]. It was not until the 1990s that the Internet grew faster [42] and was made available to the German non-academic population [43]. Consequently, people born in the 1990s grew up in a much more digitalized world than people born way before the 1990s. In 2013, a finding was similar to this suggestion: people born after 1980 reported higher Internet anxiety and lower Internet identification than those born after 1993 [44]. Therefore, one could expect that a younger generation may feel more open, trusting, and competent regarding the usage of digital media (i.e., weight management applications). Contrary to this, an older generation might prefer face-to-face interventions. Previous research has also shown that the willingness to use eHealth applications depends on several aspects [45]. Overall, for example, in clinical and dietetic settings, the following aspects are identified as critical: access to the technology, standardization, attitude (including the knowledge about the benefits and the appreciation of the need and willingness to use technology), aptitude (skills and training to use technology), and advocacy in supporting the initiative [45]. Accordingly, the age effect detected in the present study can be explained by considering these aspects [45] and the generation-dependent report regarding Internet anxiety and Internet identification [44].

However, gender, the degree of obesity, and previous experience with eHealth interventions were not related to the acceptance. Focusing on weight control behaviors, in a previous study, the prevalence among women was higher than among men, but the types of behaviors used and the duration and consistency of their use did not show any significant differences [46]. The finding that the level of obesity does not influence the acceptance of weight management applications seems relatively counterintuitive at first. In a previous study, there was an association between the degree of adiposity and participation in sports for both genders [47]. On closer inspection, however, one should reconsider that (1) about 50% of all participants described their weight as appropriate and (2) the estimation of relative body weight was found to be inaccurate [47]. Consequently, one might assume the acceptance of weight management does not necessarily flourish from a high degree of obesity. Generally speaking, satisfaction with one’s weight is a subjective feeling, so the desire to lose weight can exist in underweight, normal, and overweight people. At first, one might assume that eHealth interventions are very similar. This mainly applies to the following characteristics: (1) goal setting and (2) monitoring diet and activity [9,48,49]. At best, they also contain a social media component that suggests a sense of community [9,48,49]. That previous experience with eHealth interventions was not related to the acceptance of weight management applications could be explained by the technology’s characteristics. Firstly, as mentioned above, technical progress is a rapidly progressing process [11,43]. Accordingly, eHealth interventions are constantly being further developed and changed. Secondly, there is a whole range of eHealth interventions (e.g., SMS, websites, and smartphone applications) [11]. Someone who has already participated in such an eHealth intervention beforehand may have just as much experience in using the chosen tool as a so-called bloody beginner.

To date, multiple studies confirmed a positive relation between the so-called original UTAUT model (including PE, EE, SI) and the acceptance of eHealth applications [20,21,24]. Congruent with these findings, all three predictors of the original UTAUT model could explain 60% (overall model: 61.2%) of the variance in acceptance in the current study. Besides these UTAUT predictors, psychometric and eHealth-related data could be identified as significant predictors. In detail, eating disorder symptoms, confidence with social media, as well as Internet anxiety are critical predictors of acceptance—but only without the UTAUT predictors’ influence. While eating disorder symptoms and confidence with social media encourage acceptance, Internet anxiety diminishes it and represents an important barrier. This finding also fits with two previously mentioned findings, which at the same time form explanatory approaches. Firstly, the acceptance of weight management applications might not necessarily flourish from a high degree of obesity. Generally speaking, satisfaction with one’s weight is a subjective feeling, so the desire to lose weight can exist in underweight, normal, and overweight people (see [47]). The basic prerequisite for using such weight management applications depends on each participant’s desire to lose weight and motivation for change. Since people who suffer from an eating disorder are often not satisfied with their weight and cannot control their eating behavior [50,51,52], the positive influence of such symptom burdens on acceptance is intuitive. Secondly, previous studies show that insecurity or distrust regarding the Internet may impact the acceptance of Internet-supported weight management applications [44] as well as eHealth interventions in general [21]. That Internet anxiety decreases acceptance further supports these findings.

However, compared to the original UTAUT model, the extended UTAUT model (including sociodemographic, medical, psychometric, and eHealth-related data) could not explain more of the variance. This is in contrast to previously published findings [35].

Taking the age-effect together with the factors of an extended UTAUT model, one might conclude that acceptance depends individually on the target group (old vs. young), modality of the technology (mobile app, television, or PC program), and thematic focus (e.g., weight) of the eHealth intervention. For example, in 2016, scientists showed that older generations (i.e., 50 years and above) tend to be easier to reach via conventional media (i.e., home telehealth services) [41]. An extended UTAUT model with six predictors was tested, which was able to explain a total of 77% of the total variance explained in the acceptance [41]. The acceptance of the so-called home telehealth services could be optimized considering PE, EE, facilitating conditions (technical support available), doctor’s opinion (feeling of security of an objective and validated source of information), computer anxiety, and perceived security (i.e., doctor’s opinion diminishes the perceived risk) [41]. The SI seems to be rather irrelevant, especially for older people [41].

Knowing about considerable factorial individuality and diversity allows one to define the direction and focus of future research. This could be relevant to test future extended UTAUT models and maximize individually tailored eHealth management interventions regarding their general acceptance in the respective target group. In order to achieve this acceptance maximization, it is necessary to identify both relevant and irrelevant factors. Ultimately, this knowledge provides the basis for an optimization process.

### Limitations

In the following, some limitations of the present study are discussed, which have to be considered when interpreting the results.

Firstly, this is an online survey. This means that the recruitment of the subjects depends on their Internet access. Accordingly, one could assume a selection bias. In detail, test subjects who have Internet access and the necessary know-how to deal with it took part in this study. Furthermore, the present sample has an unequal gender distribution (89.3% female vs. 10.7% male), making it impossible to examine a gender effect. The overrepresentation of some parts of the population speaks against the representativeness and generalizability of the study. However, this is not a rare problem in psychological studies. In detail, 96% of psychological studies are conducted on WEIRD samples (i.e., Western, educated, industrialized, rich, and democratic) [53]. This bias seems to apply to 99% of the studies, reducing their generalizability and external validity [54]. Therefore, overrepresentation is an undesirable but quite common bias in psychological research.

Another limitation is the so-called intention-behavior gap, “which describes the failure to translate intentions into action” [55]. Transferred to the present study, the online survey only measured the BI but not the observable behavior. Accordingly, one can assume that not all subjects with BI actually showed the desired behavior. In percentage terms, the variation in health behavior is assumed to be 30% to 40% [56,57]. In order to elucidate this still unknown intention–behavior gap, future research would have to examine, for example, on a behavioral level, how many people with BI show the desired behavior afterward.

In general, the online survey was based on self-report, which might present an additional limitation. However, the aspect of a distorted self-perception is particularly relevant given that studies on the subject of overweight/obesity have detected an inaccuracy concerning the estimation of relative body weight [47]. Overall, using self-report was mandatory, and anonymization might minimize its biases. Taken together, all of these limitations provide a knowledge base that offers potential for improvement in future research.

## 5. Conclusions

The present study was able to identify predictors of the acceptance of weight management applications in an overweight/obese sample. Overlapping with previous research, the PE, EE, and SI (UTAUT factors) were identified as core predictors for acceptance in this target group. Overall, the acceptance of weight management applications in overweight and obese people was high. Acceptance, in particular, showed an apparent age effect, meaning that younger individuals showed a higher level of acceptance. Although no better acceptance prediction could be achieved using an extended UTAUT model, using descriptive sample values could still generate valuable knowledge. Given the dramatically increasing numbers of overweight and obesity, such knowledge generation is essential for optimizing and, ultimately, succeeding with weight management applications.

This optimization and implementation generally lead to accepting such weight management applications. The present study contributes to the knowledge base in the same métier but should be supplemented by further research. The scientifically based knowledge should be used in order to increase the acceptance of weight management applications developed in the future.

## Figures and Tables

**Table 1:** Sociodemographic and medical characteristics.

Characteristics	Participants	
	*N*	%
**Sex**		
Female	392	89.3
Male	47	10.7
**Age (in years)**		
18–34	75	17.1
35–44	133	30.3
45–54	136	31.0
>55	95	21.6
**Marital status**		
Single	79	15.9
Married	230	52.4
In a relationship	78	17.8
Divorced/separated	46	10.5
Widowed	9	2.1
Other	6	1.4
**Educational level**		
University education	63	14.4
Higher education entrance qualification	93	21.2
Intermediate secondary education	198	45.1
Lower secondary education	76	17.3
No qualification	3	0.7
Other	6	1.4
**BMI categories**		
Overweight	66	15.0
Obesity grade I	67	15.3
Obesity grade II	72	16.4
Obesity grade III	234	53.3
**Mental disorder**		
Yes	159	36.2
No	280	63.8
**Occupational status**		
Employed	301	68.6
Unemployed	138	31.4

Note. Total *N* = 439.

**Table 2:** Differences in acceptance (UTAUT behavioral intention scale) by sociodemographic and medical data.

Variable	*N*	%	Mean (*SD*)	Test	*p*-Value
**Sex**				*t*_437_ = 1.19	0.236
Female	392	89.3	3.84 (0.92)		
Male	47	10.7	3.67 (1.04)		
**Age (in years)**				*F*_3435_ = 5.77	0.001 ***
18–34	75	17.1	3.87 (0.79)		
35–44	133	30.3	4.07 (0.83)		
45–54	136	31.0	3.62 (1.03)		
>55	95	21.6	3.74 (0.95)		
**BMI categories**				*F*_3435_ = 2.10	0.100
Overweight	66	15.0	3.71 (0.96)		
Obesity grade I	67	15.3	3.69 (1.06)		
Obesity grade II	72	16.4	4.04 (0.77)		
Obesity grade III	234	53.3	3.83 (0.93)		
**Outpatient psychotherapy**				*t*_437_ = 1.35	0.651
No	355	80.9	3.85 (0.92)		
Yes	84	19.1	3.70 (0.97)		
**Educational level**				*F*_5433_ = 0.44	0.821
University education	63	14.4	3.75 (0.92)		
Higher education entrance qualification	93	21.2	3.85 (0.96)		
Intermediate secondary education	198	45.1	3.87 (0.89)		
Lower secondary education	76	17.3	3.73 (1.03)		
No qualification	3	0.7	3.67 (1.15)		
Other	6	1.4	4.06 (0.53)		
**Mental disorder**				*t*_437_ = 1.28	0.749
Yes	159	36.2	3.75 (0.96)		
No	280	63.8	3.87 (0.91)		
**Occupational status**				*t*_437_ = −0.22	0.826
Employed	301	68.6	3.82 (0.91)		
Unemployed	138	31.4	3.84 (0.97)		

Note. Total *N* = 439. *** *p* < 0.001. The mean comparisons were carried out using both *t*-tests and ANOVAs. The α values were corrected via post hoc tests using the Bonferroni method. The DV acceptance of weight management applications was measured via the UTAUT BI (Behavioral Intention) scale.

**Table 3:** Summary of hierarchical regression model for variables predicting acceptance of weight management applications (extended UTAUT model).

Predictor	*β*	*B*	*T*	*p*-Value	*R²*	*Δ R²*
**Step 1: Sociodemographic and medical data**					0.02	0.02
Age	−0.07	−0.01	−1.55	0.120		
Sex	−0.06	−0.19	−1.27	0.200		
BMI	0.07	0.01	1.37	0.170		
Mental disorder	−0.08	−0.08	−1.58	0.110		
Occupational status	0.03	0.05	0.49	0.620		
**Step 2: Psychometric data**					0.05 **	0.03 ***
EDI-2-B Sum Score	−0.10	−0.02	−1.72	0.086		
PHQ-8 Sum Score	0.06	0.01	0.91	0.361		
EDE-Q8 Total	0.19 **	0.12	3.05	0.003		
**Step 3: eHealth related data**					0.09 ***	0.04 ***
Internet-induced stress	0.10	0.07	1.96	0.051		
Internet anxiety	−0.10	−0.13	−2.00	0.047 *		
Experiences with online interventions	0.08	0.15	1.63	0.105		
Confidence with social media	0.13	0.13	2.59	0.001 **		
**Step 4: UTAUT predictors** ^1^					0.61 ***	0.52 ***
UTAUT PE	0.22	0.23	5.64	<0.001 ***		
UTAUT EE	0.39	0.43	9.95	<0.001 ***		
UTAUT SI	0.32	0.40	8.59	<0.001 ***		

Note. Total *N* = 439. * *p* < 0.05, ** *p* < 0.01, *** *p* < 0.001. *β*, standardized coefficient beta; *B*, unstandardized coefficient beta; *R*², determination coefficient; *Δ R²*, changes in *R²*; *p* from model comparison using ANOVA. In steps 2, 3, and 4, only the newly included variables are presented. The DV acceptance of weight management applications was measured via the UTAUT BI (Behavioral Intention) scale. ^1^ UTAUT predictors (PE, performance expectancy; EE, effort expectancy; SI, social influence).

## Data Availability

The data presented in this study are available from the corresponding author upon reasonable request.

## Supplementary Materials

The following supporting information can be downloaded at: https://www.mdpi.com/article/10.3390/nu14091968/s1, Supplement Materials I: Translated items of the UTAUT questionnaire used in this study.

## Abbreviations

BMI	Body Mass Index
DV	Dependent Variable
EDE-Q8	Eating Disorder Examination—Questionnaire 8
EDI-2-B	Eating Disorder Inventory-2—Bulimia
IV	Independent Variable
PHQ-8	Patient Health Questionnaire-8
UTAUT	Unified Theory of Acceptance and Use of Technology
(UTAUT) BI	Behavioral Intention (Acceptance)
(UTAUT) EE	Effort Expectancy
(UTAUT) PE	Performance Expectancy
(UTAUT) SI	Social Influence
VIF	Variance Inflation Factor
