# Supplementary material for: Determinants of Acceptance of Weight Management Applications in Overweight and Obese Individuals: Using an Extended Unified Theory of Acceptance and Use of Technology Model
Source: Nutrients. 2022 May 8;14(9):1968. doi: 10.3390/nu14091968 (PMC9101973; doi:10.3390/nu14091968)
Supplement: Supplementary file 1 [file nutrients-14-01968-s001.zip › nutrients-1704645-supplementary.pdf]

nutrients

MDPI

Supplementary Material

Table S1. Supplement Materials I: Translated items of the UTAUT questionnaire used in this study.

Instruction	German	English Translation
	Bitte bewerten Sie im Folgenden jede Aussage, inwieweit Sie dieser zustimmen oder nicht zustimmen.	In the following, please rate for each statement to what extent you agree or disagree.
**Items**	**German**	**English Translation**
	1. Ich würde gerne eine Gewichts-Management App ausprobieren.	I would like to try a weight management app.
	2. Ich würde eine Gewichts-Management App nutzen, wenn es mir angeboten würde.	I would use a weight management app if offered.
	3. Ich würde eine Gewichts-Management App meinen Freunden empfehlen.	I would recommend a weight management app to my friends.
	4. Personen, die mir nahe stehen würden die Nutzung einer Gewichts-Management App gut heißen.	People close to me would approve of using a weight management app.
	5. Mein Hausarzt würde die Verwendung einer Gewichts Management App gut heißen.	My family doctor would approve of using a weight management app.
	6. Meine Freunde würden eine Gewichts-Management App gut heißen.	My friends would approve of a weight management app.
	7. Eine Gewichts-Management App könnte mein allgemeines Wohlbefinden verbessern.	A weight management app could improve my overall well-being.
	8. Eine Gewichts-Management App könnte mir bei Stress helfen.	A weight management app could help me with stress.
	9. Eine Gewichts-Management App könnte mir helfen, meine persönliche (psychische) Gesundheit zu verbessern.	A weight management app could help me improve my personal (mental) health.
	10. Die Nutzung einer Gewichts-Management App wäre keine zusätzliche Last für mich.	Using a weight management app would not be an additional burden for me.
	11. Eine Gewichts-Management App wäre einfach zu bedienen und zu verstehen.	A weight management app would be easy to use and understand.
	12. Eine Gewichts-Management App könnte ich in meinen Alltag einbauen.	I could incorporate a weight management app into my everyday life.
**answer options**	**German**	**English Translation**
	1. stimme gar nicht zu	I fully disagree
	2. stimme eher nicht zu	I tend to disagree
	3. weiß nicht	I don't know
	4. stimme eher zu	I tend to agree
	5. stimme voll zu	I fully agree

Note. Shown above is the modified UTAUT questionnaire [1] on weight management apps used in this study. Since the online survey was conducted with a German-speaking sample, the original online survey content is listed in the German column, while the respective translations are listed in the English Translation column for clarity.

References

1. Venkatesh; Morris; Davis. User Acceptance of Information Technology: Toward a Unified View. *MIS Quarterly* **2003**, *27*, 425, doi:10.2307/30036540.
